# Effective Double Electron Transport Layer Inducing Crystallization of Active Layer for Improving the Performance of Organic Solar Cells

**DOI:** 10.3390/nano12010015

**Published:** 2021-12-22

**Authors:** Ping Li, Lijia Chen, Xiaoyan Hu, Lirong He, Zezhuan Jiang, Minghao Luo, Haishen Huang, Wei Yuan, Yinghu He

**Affiliations:** 1 School of Physics and Electronic Science, Zunyi Normal University, Zunyi 563006, China; XY1005499@126.com (X.H.); helirong9527@126.com (L.H.); zezhuanJ@126.com (Z.J.); lmh18311641321@126.com (M.L.); haishenh@yeah.net (H.H.); hyhv@sina.com (Y.H.); 2 College of Physics and Electronic Engineering, Chongqing Normal University, Chongqing 401331, China; ljchen01@cqnu.edu.cn; 3 Department of Rail Transportation Engineering, GuiZhou Communication Polythechnic, Guiyang 551400, China; windyuanwei@126.com

**Keywords:** organic solar cell, electron transport layer, LiCl, PEIE

## Abstract

Interface modification plays an important role in enhancing the photoelectric conversion efficiency and stability of organic solar cells. In this work, alkali metal lithium chloride (LiCl) was introduced between indium tin oxide and polyethyleneimine ethoxylate (PEIE) to prepare a double-layer electron transport layer. Results show that the introduction of LiCl has dual functions. The first function is that LiCl can enhance conductivity, thereby facilitating charge collection. The second function is that the double-layer electron transport layer based on LiCl can induce the crystallization of active layer, thereby enhancing charge transport. Devices with LiCl/PEIE double layer achieve a high power conversion efficiency (PCE) of 3.84%, which is 21.5% higher than that of pristine devices (the PCE of pristine devices with pure PEIE interface layer is 3.16%).

## 1. Introduction

Organic solar cells (OSCs) have attracted the attention of many researchers due to their advantages, such as flexible large-scale production and low fabrication costs. In recent years, the reported power conversion efficiency (PCE) of single-junction OSCs has exceeded 18% [1,2]. However, OSCs have low conversion efficiency and poor stability compared with silicon-based solar cells, which is one of the main reasons that the commercial development of OSCs is limited [3]. Many methods, such as designing new device structure, synthesizing new materials, improving manufacturing processes, and interface modification, were used to improve the performance of OSCs. Interface modification plays an important role in the long-term stability and the improvement of the PCE of the device [4,5,6,7]. Lin et al. used WS_2_ rather than PEDOT:PSS to modify the interface of a device, and the PCE of the modified device is as high as 17.3% [8]. An appropriate interface modification material is extremely important for the preparation of efficient and stable OSCs. Currently, metal oxides, such as titanium oxide (TiO_X_), zinc oxide (ZnO), and stannic oxide (SnO_2_), are commonly used as charge transport materials [9,10,11,12,13]. However, these materials can cause some defects during the preparation of interface layer due to their inherent properties. For example, metal oxides cannot form a uniform thin film with a smooth surface on indium tin oxide (ITO) due to their insolubility in solution, thereby increasing charge recombination loss and leakage current and leading to a poor efficiency of the device. To address these issues, researchers proposed to use high solubility materials rather than low solubility interface materials. Polyethyleneimine ethoxylated (PEIE) is a polymer material that can be well-dispersed in the solution and is widely used in OSCs and organic light-emitting diode devices [14]. PEIE can fabricate a uniform and smooth film on ITO. However, PEIE is an insulating material, thereby hindering charge transportation. A double-layer charge transport layer prepared using two different types of materials is proposed as an electron transport layer (ETL) in OSC. Yu et al. used ZnO:PEIE composites with different PEIE concentrations as the double-layer ETL of OSC. Compared with pure ZnO as the ETL, the PCE of a device with double-layer ETL is effectively increased from 3.01% to 3.52% [15]. Kim et al. reported that a PCE of 8.21% is obtained for a device with graphene and polymer PEIE as a double ETL based on PTB7:PC_71_BM active materials [16].

Alkali metal lithium chloride (LiCl) has good electrical conductivity and is widely used in perovskite solar cells and OSCs [17,18,19]. Ling et al. combined LiCl with ZnO as an ETL, and a PCE of 3.62% for polymer solar cells based on the poly (3-hexylthiophene) and [6,6]-phenyl-C61-butyric acid methyl ester (P3HT:PC61BM) system was obtained [17]. Sheng et al. added LiCl in the perovskite solution, which effectively increases the conductivity of the device, and the efficiency of the device is significantly improved [19]. They indicated that Li ions can enhance the conductivity in perovskite solar cell and promote the crystallization of perovskite film [20,21]. By taking advantage of the conductivity of Li ions, the combination LiCl and PEIE with insulating properties is expected to improve the conductivity of the interface modification layer. Relevant studies have not been reported. In this work, LiCl and PEIE were combined as a double-layer ETL to fabricate OSCs on the basis of P3HT:PC61BM. A high PCE of 3.84% was achieved for the device with LiCl/PEIE double layer. The effect of the introduction of LiCl on the performance of OSCs and the mechanism process was studied.

## 2. Materials and Methods

### 2.1. Materials

The ITO glass substrates were purchased from Shenzhen Huanan Xiangcheng Technology Co., Ltd. (Shenzhen, China) (transmittance ≥ 89%), and anhydrous 2-methoxyethanol (99.8%) and polyethyleneimine (PEIE, 37%) were purchased from Sigma Aldrich (St. Louis, MO, USA). PCBM, P3HT, thieno[3,4-b] thiophene/benzodithiophene (PTB7), [6,6]-phenyl C_71_-butyric acid methyl ester (PC_71_BM) and LiCl (99.5%) were purchased from J&K Chemicals (Beijing, China). Metallic silver (Ag, 99%), molybdenum trioxide (MoO_3_, 99%), and bath copper spirit (BCP, 99%) were provided by Xi’an Polymer Light Technology Corp (Xi’an, China).

### 2.2. Device Preparation

The ITO glass substrates were continuously and ultrasonically cleaned with cleaning agent, absolute ethanol, acetone, and isopropanol for 20 min. The devices were dried with nitrogen and underwent plasma treatment. The OSCs with the structure of ITO/PEIE or X-LiCl-PEIE/P3HT:PCBM/MoO_3_/Ag were fabricated, where X represents LiCl aqueous solutions with concentrations of 0.5, 1, 2, 5, and 10 mg/mL, which were marked as pure PEIE, 0.5-LiCl-PEIE, 1-LiCl-PEIE, 2-LiCl-PEIE, 5-LiCl-PEIE, and 10-LiCl-PEIE, respectively. For the fabrication of the devices, the LiCl aqueous solution with various concentrations was spin-coated on the surface of the treated ITO at 2000 rpm for 40 s and then thermal annealed in air at 120 °C for 20 min. After cooling to room temperature, a certain concentration of PEIE solution was spin-coated on the surface of the LiCl film at 4000 rpm for 60 s and then thermal annealed in air at 120 °C for 15 min to obtain the LiCl/PEIE double-layer ETL. After cooling, the ITO glass substrates with ETL were transferred in a glovebox for fabricating the active layer. The P3HT:PCBM solution (35 mg/mL of concentration, P3HT:PCBM = 18:17) and PTB7:PC_71_BM solution (25 mg/mL, PTB7:PC_71_BM = 1:1.5) were spin-coated on the prepared ETL at a speed of 870 r/min for 40 s. The samples with active layer were then transferred to the growth chamber of high-vacuum thermal evaporation. A MoO_3_ thin film with a thickness of 6 nm and a Ag film with a thickness of 60 nm were grown at 0.01 and 0.05 nm/s as a hole transport layer and an electrode, respectively. During the growth process of MoO_3_ and Ag electrodes, the vacuum degree of the growth chamber was controlled below 1 × 10^−4^ Pa. Shadow mask was used during thermal evaporation to define the active area of 0.09 cm^2^ for the devices. The pristine device was a pure PEIE as an ETL and was marked as pure PEIE device.

### 2.3. Characterization of Films and Inverted OSCs

The current–voltage (I–V) characteristics of the devices were measured by using 100 mW/cm^2^ simulated sunlight AM 1.5G and a Keithley 2400 SourceMeter purchased from Zuoli (Beijing, China). Electrochemical impedance spectroscopy (EIS) was measured using a CS Series electrochemical workstation purchased from Wuhan Kesite Company (Wuhan, China). The conductivity of devices was evaluated using Keithley 2400. X-ray diffraction (XRD) patterns were obtained using an XRD with Cu Kα 1 radiation (λ¼1.5406), with generation power of 40 kV tube voltage and 40 mA tube current operated in the locked couple mode. The morphology of the active layer was characterized through atomic force microscopy (AFM). The absorption spectrums of the active layers were measured with an ultraviolet–visible (UV–vis) photometer.

## 3. Results and Discussion

**Figure 1:**
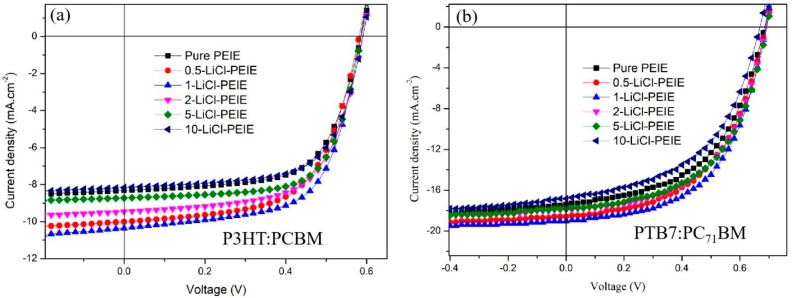
*I*–*V* curves of OSCs based on different ETLs, (**a**) P3HT:PCBM-based devices and (**b**) PTB7:PC_71_BM-based devices.

Pure PEIE and X-LiCl/PEIE (where X is 0.5, 1, 2, 5, and 10 mg/mL) were used as an ETL. OSC devices with the structure of ITO/ETL/P3HT:PCBM/MoO_3_/Ag were fabricated and marked as pure PEIE, 0.5-LiCl-PEIE, 1-LiCl-PEIE, 2-LiCl-PEIE, 5-LiCl-PEIE, and 10-LiCl-PEIE, respectively. All devices were characterized using a solar simulator with 1 sun AM 1.5 G illumination (100 mW·cm^−2^). The I–V curves of the OSCs based on different ETLs are depicted in Figure 1a. The performance parameters of the OSCs, including short-circuit current (*J_sc_*), open-circuit voltage (*V_oc_*), fill factor (*FF*), and PCE are summarized in Table 1. The devices of the best PCEs were used for the following discussion. With only PEIE as ETLs (pure PEIE), the device yields a PCE of 3.19%, *J_sc_* of 8.32 mA·cm^−2^, *V_oc_* of 0.58 V, and *FF* of 65.5%. The performance of the OSCs is remarkably enhanced by inserting LiCl between ITO and PEIE as ETLs. *J_sc_* increases from 8.32 mA·cm^−2^ to 10.36 mA·cm^−2^ when the LiCl concentration increases from 0 mg/mL to 1 mg/mL and then constantly reduces to 8.16 mA·cm^−2^ when the LiCl concentration increases to 10 mg/mL. Highest PCE of 3.84%, *J_sc_* of 10.36 mA·cm^−2^, *V_oc_* of 0.58 V, and *FF* of 62.8% are obtained by the device with 1-LiCl-PEIE as ETL. Compared with the pristine device, the PCE of the device with 1-LiCl-PEIE ETL improves by 21.5%. The enhancement in PCE is mainly contributed by the increasing *J_sc_*, which is consistent with the smallest *R_s_* of 7.5 Ω.cm^2^ and largest *R_sh_* of 1982 Ω·cm^2^. The same trend was observed by the PTB7:PC_71_BM-based devices and shown in supporting information (Figure 1b). With only PEIE as ETLs, the PTB7:PC_71_BM-based device yields a PCE of 6.11%, *J_sc_* of 17.27 mA cm^−2^, *V_oc_* of 0.69 V, and *FF* of 51.2%. The highest PCE of 7.33%, *J_sc_* of 19.07 mA cm^−2^, *V_oc_* of 0.69 V, and *FF* of 55.4% are obtained by the PTB7:PC_71_BM-based device with 1-LiCl-PEIE as an ETL. Compared with the pristine device, the PCE of the device with 1-LiCl-PEIE ETL improves by 19.6%. The result indicates that the introduction of LiCl may reduce the interface contact resistance and charge recombination loss, thereby enhancing the charge transport ability and improving the *J_sc_* of device with LiCl/PEIE as an ETL.

**Figure 2:**
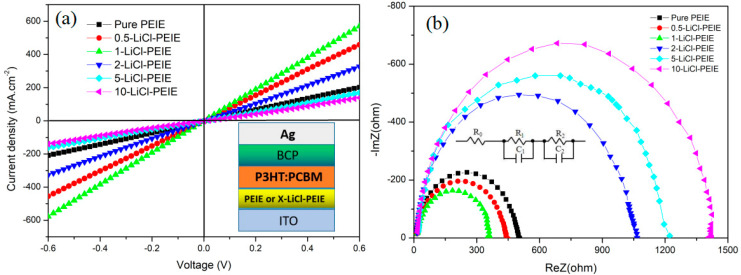
(**a**) I–V curves of the device with the structure of ITO/PEIE (X-LiCl/PEIE)/P3HT:PCBM/BCP/Ag. (**b**) EIS of P3HT:PCBM-based OSCs with different LiCl/PEIE interfacial layers.

The I–V curves of the device with the structure of ITO/PEIE (or *X*-LiCl/PEIE)/P3HT:PCBM/BCP/Ag are recorded to understand the enhancement of *J_sc_* from the contribution of the LiCl/PEIE double layer, as shown in Figure 2, where the values of *X* are 0.5, 1, 2, 5, and 10 mg/mL. The slope of the I–V curves for the device corresponds to the conductivity of the ETL films. The slope of the device with LiCl increases with the increase in LiCl concentration, and the largest slope is achieved at 1 mg/mL of LiCl. With the increase in LiCl concentration, the slope gradually decreases. The smallest slope is achieved at 10 mg/mL of LiCl. This result is consistent with the I–V measurement in Figure 1. This finding indicates that the LiCl/PEIE double layer with 1 mg/mL LiCl has the optimal conductivity ability, and high concentration of LiCl leads to the reduction in conductivity. The EIS of the OSCs based on P3HT:PCBM is measured in the light state to confirm whether the conductivity of LiCl/PEIE as an ETL can be increased and the resistance at the interface between ITO and the active layer can be reduced, as shown in Figure 2b. These experimental results can be well-fitted by using the equivalent circuit shown in the inset of Figure 2b, as shown in Table 2, where *R*_0_, *R*_1_, and *R*_2_ are the series resistance, transfer resistance in ETL, and charge recombination resistance at the interface between ETL and the active layer, respectively [22,23]. As shown in Table 2, the smallest *R*_0_ of this device is achieved. This result indicates that the enhancement in conductivity is attributed to the formation of ohmic contact between the LiCl/PEIE and the active layer. The smallest values of *R*_1_ and *R*_2_ are found for the best performing device with 1-LiCl/PEIE ETL, which are 50.78 and 170.17 Ω, respectively. These results indicate that the insertion of a certain amount of LiCl between PEIE and ITO can enhance the charge transfer rate and reduce the recombination at the interface between the ETL and ITO. However, LiCl/PEIE ETL with high LiCl concentration increases the interface resistance, thereby improving charge recombination and leading to photocurrent reduction. The largest charge recombination of the device with 10-LiCl/PEIE ETL found by EIS measurements is consistent with the smallest *J_sc_* of this device in Figure 1a.

**Figure 3:**
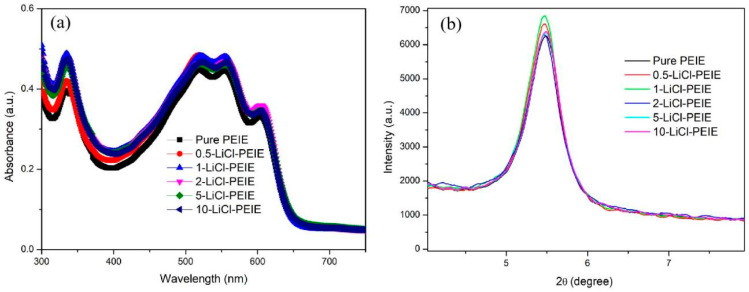
(**a**) Absorbance spectrum and (**b**) XRD of P3HT:PCBM film with various ETLs.

Considering the contribution of absorption to the photocurrent, two half-cells with the structure of ITO/PEIE/P3HT:PCBM and ITO/*X*-LiCl/PEIE/P3HT:PCBM were fabricated to measure the light absorption of the P3HT:PCBM active layer, where the values of *X* are 0.5, 1, 2, 5, and 10 mg/mL. As shown in Figure 3a, the absorption of P3HT:PCBM film with LiCl/PEIE is slightly higher than that of the active layer film with pure PEIE. When the LiCl aqueous solution is 1 mg/mL, the light absorption is the highest, which is consistent with the largest *J_sc_* of this device in Figure 1a. This enhancement in light absorption may be ascribed to the P3HT:PCBM crystallization induced by introducing LiCl. An XRD analysis of the P3HT:PCBM film with PEIE and 1-LiCl was conducted to confirm whether the LiCl induces P3HT:PCBM crystallization, as depicted in Figure 3b. A peak around 5.4° can be observed in all films, which is assigned to the crystallographic plane (100) of P3HT crystals [24]. The diffraction intensity of P3HT:PCBM films with LiCl/PEIE is higher compared with a film with pure PEIE, indicating that more crystalline P3HT is formed in films with LiCl/PEIE (Figure 3b). These results indicate that the P3HT:PCBM crystallization can be induced by introducing LiCl.

**Figure 4:**
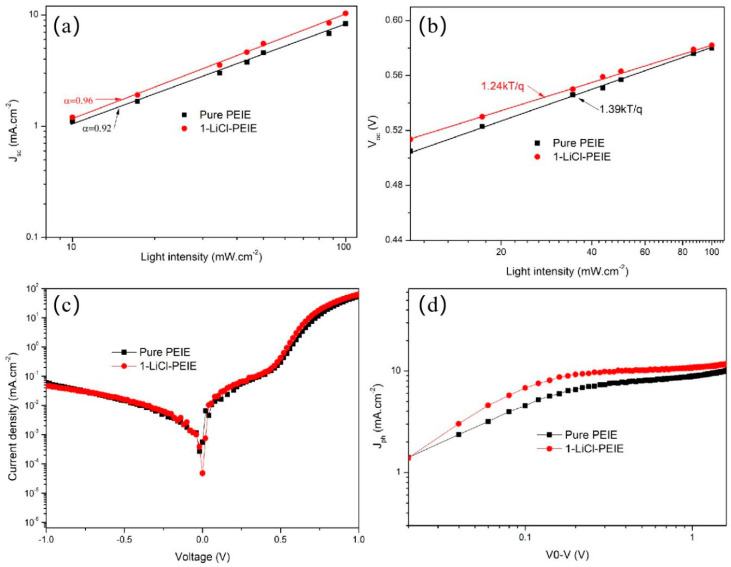
(**a**) Light intensity dependence of short circuit current (*J_sc_*), (**b**) light intensity dependence of open-circuit voltage (*V_oc_*), (**c**) I–V curves in the dark, (**d**) photocurrent (*J_ph_*) measurement for devices with pure PEIE and 1-LiCl-PEIE ETLs.

To further understand the charge recombination characteristics, the light intensity dependent *J_sc_* and *V_oc_* characteristics for the device with pure or 1-LiCl-PEIE were measured and shown in Figure 4. An α value of 0.96 was obtained for the device with 1-LiCl-PEIE. The relatively small α value was determined to be 0.92 for the device with pure PEIE, suggesting the LiCl-based double layer reduces the bimolecular recombination (Figure 4a). The reduction in bimolecular recombination can be attributing to the decrease in defect for P3HT:PCBM film. The defect can create the trap state. The trap state will result in the charge recombination. The slope of 1.39 kT/q for the device with pure PEIE is bigger than that of 1.24 kT/q for the device with 1-LiCl-PEIE (Figure 4b) [25]. The result hints that the LiCl-based double layer can effectively suppress the trap-assisted recombination [26], suggesting further promoting the extraction of charge. The enhancement in the extraction of charge can be confirmed by the higher rectification ratio for the device with 1-LiCl-PEIE compared with the device with PEIE (Figure 4c). The photocurrent (*J_ph_*) versus the effective voltage (*V_eff_*) was measured to understand charge extraction [25]. *J_ph_* is given by *J_ph_* = *J_L_ − J_D_*, where *J_L_* is the current density under light illumination and *J_D_* is the dark current density. *V_eff_* is given by *V_eff_* = *V*_0_ − *V*, where *V*_0_ is the compensation voltage where *J_ph_* = 0 mA cm^2^ and *V* is the applied bias voltage. A dramatic saturation regime was observed for the device with 1-LiCl-PEIE (Figure 4d), which suggests a negligible trapped charge and effective charge extraction/collection. The device with pure PEIE showed no clear saturation regime in *J_ph_* with increasing *V_eff_*, indicating significant electron-hole recombination. In addition, the photocurrent density of the device with 1-LiCl-PEIE was higher than that of the device with pure PEIE, suggesting higher charge extraction in the LiCl-based device. These results are consistent with the photovoltage characteristics, EIS, and conductivity analysis.

**Figure 5:**
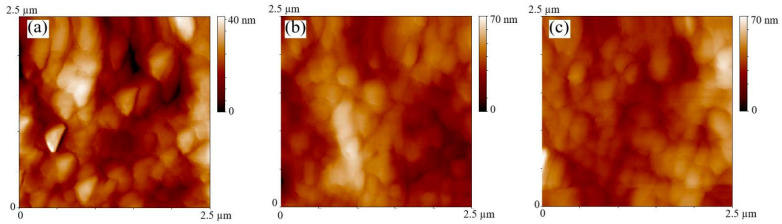
Surface morphology of the P3HT:PCBM film with PEIE (**a**) and 1-LiCl/PEIE (**b**) or 5-LiCl/PEIE ETL (**c**). The scan size is 2.5 μm × 2.5 μm.

The surface morphology of the P3HT:PCBM film with various ETLs was investigated. The AFM surface images of the P3HT:PCBM film with pure and LiCl/PEIE ETL are shown in Figure 5. The surface roughness of P3HT:PCBM film with PEIE is 5.8 nm, which is consistent with the previously reported result [27], as shown in Figure 5a. The surface roughness of the P3HT:PCBM film with 1-LiCl/PEIE and 5-LiCl/PEIE ETL is measured as 7.96 and 6.59 nm, respectively, as depicted in Figure 5b,c. The increase in roughness is related to the improvement in crystallinity of the two phases [28,29]. This condition suggests a better charge separation and charge carrier mobility in the two phases for the samples with the LiCl/PEIE interface layer. Some defects are observed on the surface of the P3HT:PCBM film with PEIE ETL. By contrast, a uniform surface is observed on the surface of the P3HT:PCBM film with LiCl/PEIE ETL. This finding indicates that the introduction of LiCl can reduce the trap state of the P3HT:PCBM film. The increase in crystallization and the decrease in defect for the P3HT:PCBM film are beneficial to promote the charge transport in an active layer, resulting in the enhancement of *J_sc_* of the device and improving the performance of OSCs.

**Figure 6:**
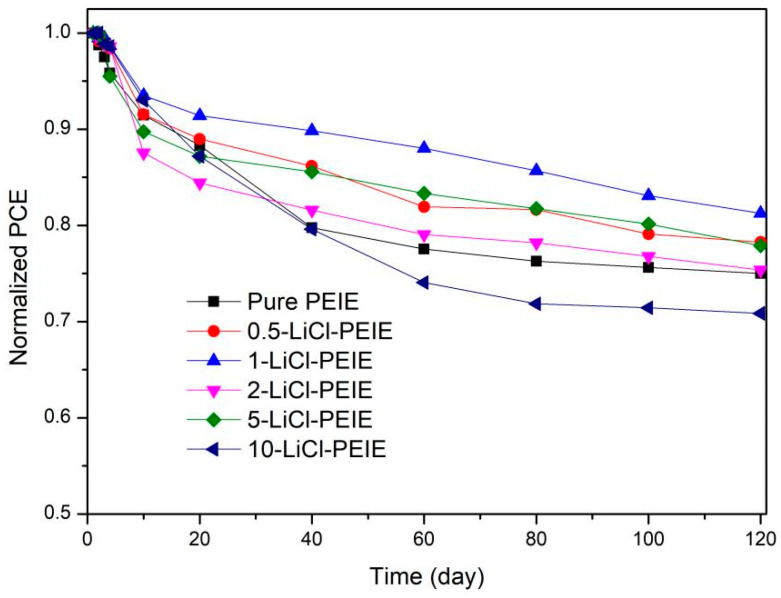
Long-term stability of the unencapsulated devices with different ETLs in N2 atmosphere with one sun illumination at 25 °C.

In addition, long-term stability of the unencapsulated devices with different ETLs in the N_2_ atmosphere with one sun illumination at 25 °C were evaluated and shown in Figure 6. After 120 days, PCE of the LiCl-based device is higher than that of the device with pure PEIE except for the 10-LiCl-PEIE device, indicating the device’s stability can be improved by a double-layer ETL.

## 4. Conclusions

In this study, an OSC with an efficiency of 3.84% was prepared using LiCl/PEIE as an ETL. An EIS analysis and conductivity measurement of the device indicate that LiCl/PEIE as the double-layer ETL and is conducive to improving the conductivity of electrons due to the reduction in the contact resistance of interface between the ITO and active layer compared with the single-layer PEIE ETL. XRD, UV–vis, and AFM characterization indicate that the introduction of LiCl is beneficial to enhancing the crystallization of the active layer film, reducing the trap state, and improving the transport of free carriers, resulting in the enhancement of *J_sc_*, and improving the performance of the device. This study proposed an effective method to fabricate the high efficiency solar cells.

## Figures and Tables

**Table 1:** Performance parameters of P3HT:PCBM-based OSCs with different ETLs. The presented value is the best one observed in this study.

Device	*V_o_* (V)	*J_sc_* (mA·cm^−2^)	*FF* (%)	*R_s_* (Ω·cm^2^)	*R_sh_* (Ω·cm^2^)	PCE (%)	
						Highest	Average ^a^
Pure PEIE	0.58	8.32	65.5	11.3	1281	3.16	3.04
0.5-LiC-PEIE	0.58	10.01	60.1	9.6	1832	3.54	3.43
1-LiCl-PEIE	0.58	10.36	62.8	7.5	1982	3.84	3.61
2-LiCl-PEIE	0.58	9.44	64.4	9.9	1652	3.53	3.21
5-LiCl-PEIE	0.58	8.72	68.2	11.7	1127	3.45	3.19
10-LiCl-PEIE	0.59	8.16	66.0	15.1	987	3.12	2.98

^a^ Average over 10 devices.

**Table 2:** EIS of OSCs based on different ETLs.

Device	*R*_0_ (Ω)	*R*_1_ (Ω)	*R*_2_ (Ω)
Pure PEIE	85.23	79.15	332.12
0.5-LiC-PEIE	73.14	68.51	285.54
1-LiCl-PEIE	62.42	50.78	170.17
2-LiCl-PEIE	130.73	127.61	433.16
5-LiCl-PEIE	178.81	153.52	489.22
10-LiCl-PEIE	245.95	331.19	888.25

## Data Availability

The data presented in this study are available on request from the corresponding author.
